# In Vivo Reporters for Visualizing Alternative Splicing of Hormonal Genes

**DOI:** 10.3390/plants9070868

**Published:** 2020-07-08

**Authors:** Ivan Kashkan, Ksenia Timofeyenko, Eva Kollárová, Kamil Růžička

**Affiliations:** 1 Laboratory of Hormonal Regulations in Plants, Institute of Experimental Botany, Czech Academy of Sciences, 165 02 Prague, Czech Republic; kashkan@ueb.cas.cz (I.K.); timofeyenko@ueb.cas.cz (K.T.); 2 Functional Genomics and Proteomics of Plants, Central European Institute of Technology and National Centre for Biomolecular Research, Masaryk University, 625 00 Brno, Czech Republic; eva.slikova@natur.cuni.cz

**Keywords:** alternative splicing, auxin, cytokinins, brassinosteroids, *AHP6*, *DWF4*, *PIN7*, D-amino acid oxidase, genetic screen, *Arabidopsis*

## Abstract

Rapid progress in plant molecular biology in recent years has uncovered the main players in hormonal pathways and characterized transcriptomic networks associated with hormonal response. However, the role of RNA processing, in particular alternative splicing (AS), remains largely unexplored. Here, using example genes involved in cytokinin signaling, brassinosteroid synthesis and auxin transport, we present a set of reporters devised to visualize their AS events in vivo. These reporters show a differential tissue-specific expression of certain transcripts and reveal that expression of some of the them can be changed by the application of the exogenous hormone. Finally, based on the characterized AS event of the PIN7 auxin efflux carrier, we designed a system that allows a rapid genetic screening for the factors upstream of this AS event. Our innovative toolset can be therefore highly useful for exploring novel regulatory nodes of hormonal pathways and potentially helpful for plant researchers focusing on developmental aspects of AS.

## 1. Introduction

Advanced sequencing has revealed a stunning complexity of eukaryotic (and plant) transcriptomes, shaped by alternative splicing (AS). In contrast to the increasing depth and refinement of the transcriptomic approaches, very few individual AS events have been experimentally characterized in detail. In multicellular organisms, a crucial feature of the AS events is the temporal and tissue-specific extent of their expression. Fluorescent AS sensors represent a powerful tool that allows for AS detection in vivo and in situ. These reporters are widely used in animal systems [1,2,3,4] and are also helpful in deciphering the upstream stimuli modifying the transcript levels inside cells [2,3,5,6,7]. Nonetheless, their usage in plant systems appears to be rare [8,9,10].

Phytohormones integrate developmental programs and environmental cues during the whole plant lifecycle. Although the key factors governing hormonal signaling, transport, and their direct internal levels have been deeply characterized, only a little work has been dedicated to the role of AS in these pathways. Though a few examples underlining the functional role of AS have been described for abscisic acid [11,12,13,14,15], knowledge about the involvement of AS in hormonal regulations of cytokinin, brassinosteroid, and auxin pathways remains sporadic [13,16,17,18,19,20].

Here, we reveal the advantage of fluorescent sensors directed for the visualization of various types of AS and illustrate their usage on several genes involved in phytohormonal processes. To this end, we examined the splice variants of the genes involved in cytokinin signaling (*AHP6* [21]), brassinosteroid synthesis (*DWF4* [22,23]), and auxin transport (*PIN4* and *PIN7* [24,25,26]), and investigated their expression in the *Arabidopsis* seedling root tip. We reveal that some of the splice isoforms are differentially expressed and their expression levels can be altered following hormonal treatment. Finally, based on the *PIN7* AS reporter, we also present a system for an efficient forward genetic screening aimed for the identification of the regulators upstream of the *PIN7* AS event.

## 2. Results

### 2.1. Reporters for Visualizing Common Types of AS Events in Plant Hormonal Genes

**Figure 1:**
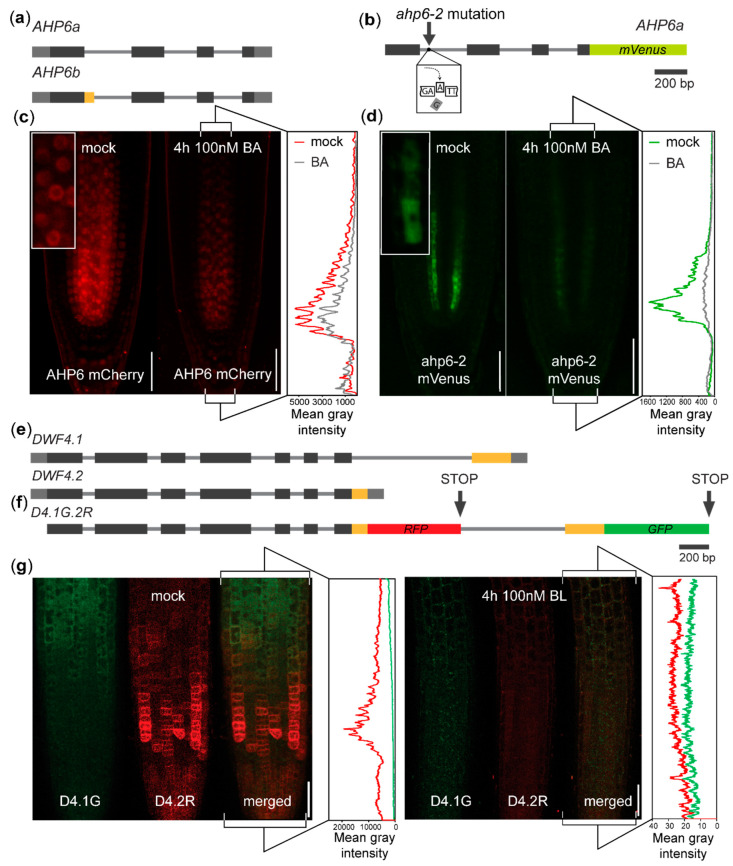
Reporters visualizing AS of *AHP6* and *DWF4* in the root tip: (**a**) Diagram of the coding regions of the *AHP6* gene. (**b**) Scheme of the *ahp6-2-mVenus* reporter for visualizing the AHP6a-mVenus isoform. (**c**) *AHP6:AHP6-mCherry* line, containing the genomic *AHP6* sequence, shows that the AHP6 protein is distributed throughout the root tip stele [21] (left); the treatment with the synthetic cytokinin benzyladenine (BA) decreases the expression of the wild type *AHP6:AHP6-mCherry*. Note the predominantly nuclear presence of the AHP6-mCherry foci (inset). (**d**) *AHP6:ahp6-2-mVenus* is present only in the protoxylem founder cells and neighboring pericycle (left); following the treatment with BA, the reporter expression decreases even more notably. Inset: a perinuclear cytoplasmic or endocompartment localization of ahp6-2-mVenus. (**e**) Diagram of the coding regions of the *DWF4* gene. (**f**) Scheme of the D4.1G.2R reporter visualizing the DWF4.1 and DWF4.2 expression. (**g**) Following the treatment with brassinolide (BL), the fluorescence signal drops below the detection limit of the microscope. Bars, 100 µm, on (**c**), (**d**) and (**g**). Black—coding regions, grey—UTRs, thin grey line—introns, yellow—regions changed by AS, red—RFP, green—GFP, on (**a**), (**b**), (**e**) and (**f**).

Previously, Mähönen and collaborators [21] found that *AHP6*, a negative regulator of cytokinin signaling, is processed into two splice variants. The amino acid sequence encoded by the canonical transcript (*AHP6b*) corresponds to that of a common AHP transducer of its class [21,27,28], while the minor *AHP6a* isoform lacks a large part of the first exon (Figure 1a). The weak *ahp6-2* allele contains a mutation leading to a cryptic AS site inside the first intron, which leads to the exclusive expression of the alternative *AHP6a* transcript [21]. To visualize the expression of the *AHP6a* isoform, we fused the *ahp6-2* sequence with the *mVenus* tag under the control of the native *AHP6* promoter (Figure 1b). Wild type AHP6-mCherry, attributable to the more abundant canonical *AHP6b* transcript [21] (Figure 1c), is synthesized in the protoxylem founder cells and the adjacent pericycle and is actively distributed within the primary root stele [21,29]. In contrast, *ahp6-2-mVenus* appears to be retained at subcellular compartments within the *AHP6* promoter [21] expression domain, and it fails to migrate from the protoxylem poles of the meristem stele (Figure 1d). Cytokinin treatment leads, similar to that of the *AHP6:GFP* promoter fusion [21], to the decrease in expression of the wild type DNA-based *AHP6:AHP6-mCherry* reporter (Figure 1c). An even stronger signal drop was seen for *AHP6:ahp6-2-mVenus* (Figure 1d). This collectively indicates that such AS reporters can also be used in plants. Moreover, AS could change the properties of the AHP6 protein, conceivably including its ability to act non-cell autonomously in the root meristem and one can also speculate whether *AHP6* splice isoforms can be differentially regulated at the post-transcriptional level.

Detecting the expression of the minor AHP6a isoform in vivo, we turned to another type of hormonal AS event. We selected *DWF4*, an essential component of the brassinosteroid synthesis pathway [22]. Here, the alternative *DWF4.2* variant contains a premature stop codon in the last intron retained, compared to the canonical *DWF4.1* transcript (Figure 1e) [23]. To visualize a simultaneous expression of both splice variants, we inserted the GFP and RFP tags into coding regions prior to the termination codons of *DWF4.1* and *DWF4.2*, respectively (Figure 1f). Adapting the usage from the animal field [1], we named the reporter D4.1G.2R (for DWF4.1-GFP + DWF4.2-RFP). We observed that the *D4.1G* expression is identifiable starting from the root tip transition zone and continuing to the proximal regions of the root, while the *D4.2R* signal prevails in the root meristem (Figure 1g). Although we were unable to distinguish, due to the low signal intensity, whether AS also changes the subcellular localization of the resulting protein, the patchy character of the *D4.2R* signal in the meristem suggests that it might be a short-living protein possibly linked to specific phases of the cell cycle [30]. The low stability of DWF4 was corroborated by the treatment with brassinolide (Figure 1g). This leads to a rapid decrease in the protein expression [31] and the decline in the fluorescence signal for both variants already after 4 h of treatment (Figure 1g). It suggests that AS can change the overall expression pattern of the DWF4 protein and that the in vivo reporters can be used in *Arabidopsis* for monitoring various types of AS, such as alternative donor site selection or intron retention.

### 2.2. Reporters Monitoring AS of Auxin Transporters PIN4 and PIN7

**Figure 2:**
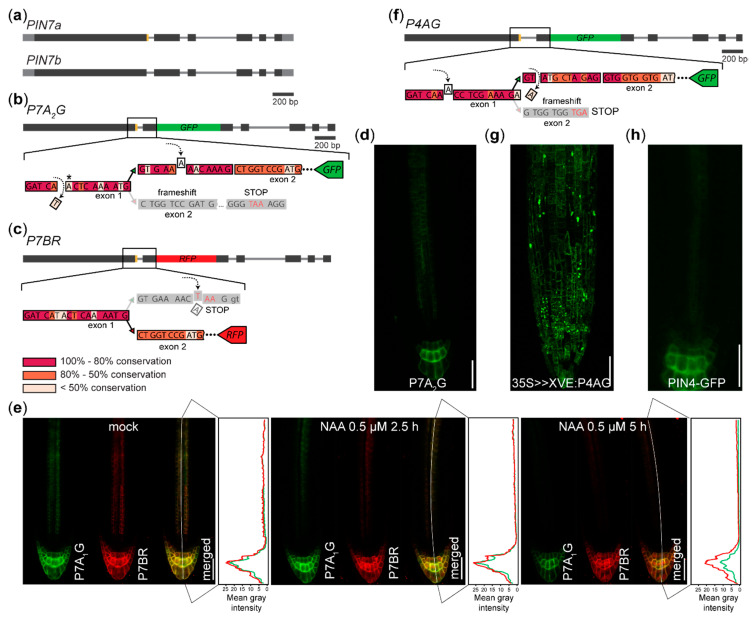
Reporters visualizing AS of *PIN7* and *PIN4* in the root tip: (**a**) Diagram of the coding regions of the *PIN7* gene. The canonical *PIN7b* isoform differs from *PIN7a* in the presence of the 12 nucleotide region (orange). (**b**) Scheme of the P7A_2_G reporter for visualizing the PIN7a isoform. Removing the nucleotide prior to the alternative splice site leads to a frameshift in the PIN7b transcript. The frame is restored by insertion of the nucleotide into the region exclusively encoded in the P7A_2_G sequence following the alternative splice site. Asterisk: a neighboring A nucleotide is removed in the P7A_1_G sensor [10] (**c**) The P7BR reporter, as outlined in [10]. The visualization of P7BR is accomplished by introducing the termination codon into the protruding region encoded entirely by PIN7a. P7AG and P7BR are encoded by two constructs that are separately transformed and then crossed. (**d**) Expression pattern of the P7A_2_G reporter. (**e**) Following the treatment with the synthetic auxin NAA, a differential decrease of expression is observed in the root columella cells, while nearly no difference in their expression change is observed in the vascular cylinder. (**f**) Scheme of the P4AG reporter for visualizing the PIN4a isoform (*PIN4* shows an identical exon/intron structure with *PIN7* [16]). Following the induction of the *35S>>XVE:P4AG* transgene, the GFP fluorescence is also seen outside (**g**) the *PIN4:PIN4-GFP* expression domain (**h**). Bars, 100 µm on (**e**), (**d**), (**g**) and (**h**). Black—coding regions, grey—UTRs, thin grey line—introns, yellow—regions modified by AS, red—RFP, green—GFP on (**a**), (**b**), (**c**) and (**f**). The color shading of the evolutional conservation code outlined (**c**) was assessed as a percentage of the preserved nucleotides on the alignment of the *PIN7*-like sequences within the *Brassicaceae* family.

We also generated the P7A_2_G (for PIN7a-*G*FP, version 2) reporter for AS of the gene encoding an auxin efflux carrier PIN7 [16,24], designed as an alternative to the P7A_1_G sensor visualizing the AS of the *PIN7a/b* event reported earlier [10] (Figure 2a–c). We observed the expression pattern and the subcellular protein localization similar to that previously reported [10] (Figure 2d), but with a lower signal intensity (data not shown). It reveals that one can design several versions of the reporters, obtaining similar results.

We treated the described P7A_1_G x P7BR (for PIN7b-RFP) AS sensor [10] with the synthetic auxin 1-naphthylacetic acid (NAA). We observed that the signal in the stele decreased after 2.5 h [32] for both PIN7 variants, while the signal in the columella persisted at nearly similar levels for P7BR, in contrast to a noticeable drop of P7A_1_G intensity after 5 h (Figure 2e). This suggests that hormonal treatment can differentially change the expression of the splice isoforms and that auxin can directly or indirectly affect gene expression also at the post-transcriptional level.

The fluorescence was also seen in the case of the P4AG (PIN4a-GFP) reporter (Figure 2f), whose expression was controlled by the β-estradiol inducible *35S* promoter [33]. Here, the P4AG signal was also present in the cells outside the native PIN4 expression domain (Figure 2g,h). These reporters can therefore also be used to test whether the expression of the splice variant is tissue-specifically predetermined.

### 2.3. A System for Genetic Screening for the Factors Upstream of the PIN7 AS Event

**Figure 3:**
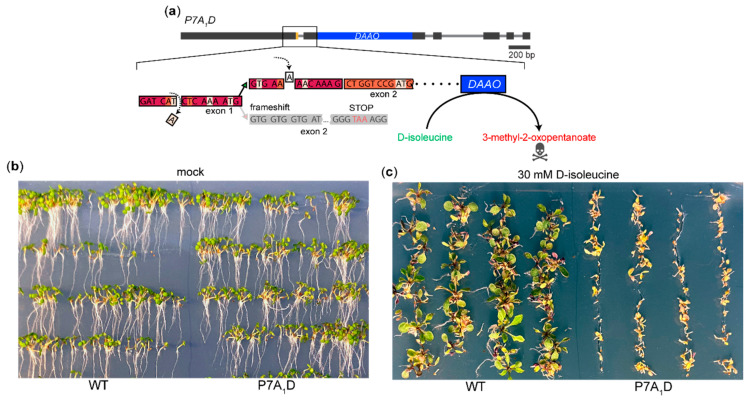
A screening system for the factors genetically upstream of the PIN7a/b splicing event: (**a**) A scheme of the P7A_1_D reporter. Physiologically inactive D-isoleucine is converted by DAAO to the toxic 3-methyl-2-oxopentanoate when P7A_1_D is expressed [37]. (**b**) The 6-days-old transgenic seedlings harboring the P7A_1_D reporter on control media. (**c**) The 12-days-old seedlings carrying the P7A_1_D reporter germinated on the media supplemented with 30 mM D-isoleucine, which leads to severe tissue lesions and loss of viability.

Although a few upstream regulators of some AS events have been identified in plants [12,13,15], no systematic effort has been made in order to explore such regulators using a classical forward genetic methodology. To design a genetic screening system for the regulators of the *PIN7a/b* AS event, we considered some of the previously proposed positive or negative selection markers, e.g., genes coding for barnase, diphtheria toxin, RNAse T1 [34,35], phosphonate monoesterase (pehA) [34,36] or D-amino acid oxidase (DAAO) [34,37,38]. DAAO, in particular, turned out to be suitable for designing such system, as the external application of the substrate can conditionally control the expected lethality of the selection marker [36,37]. We modified the P7A_1_G sensor [10] by replacing the GFP tag with DAAO [37] and generated stable lines carrying this transgene (Figure 3a).

The lines containing the *DAAO* construct termed P7A_1_D (for PIN7a-DAAO), which were indistinguishable from the wild type on the control media (Figure 3b). However, the application of D-isoleucine [37] had a dramatic impact on the seedling viability in these genotypes (Figure 3c). These experiments thus suggest that the DAAO gene can be used, along with fluorescent reporters, as a basis for a rapid genetic screen aimed for the identification of positive regulators of the PIN7 AS event.

## 3. Discussion

Here we reveal that various types of AS can be visualized in vivo and in situ in plants. As the AS reporters provide fine spatiotemporal information about the splice isoform expression, they can be highly useful in situations when quantitative (e.g., qRT-PCR) or qualitative (e.g., RNA hybridization in situ) approaches could not be reliably used. In extraordinary cases, such reporters can also extend the information delivered by the standard gene expression reporters, such as genomic DNA-based translational fusions with the reporter tag expressed only inside a subset of splice variants.

This system has certain limits, however, and a careful interpretation of isoform expression is desirable. For example, from the public transcriptomic databases, the native *DWF4.2* transcript levels in the root appear to be significantly lower than that of *DWF4.1* [23,39,40]. It can be, on the one hand, explained by the high ratio of mature versus meristematic cells present in the original RNA-sequencing experiments. On the other hand, inserting the region coding for the fluorescent tag can interfere with putative exonic elements required for the regulation of the AS event and lead to isoform expression artifact. Where possible, considering evolutional conservation is therefore highly useful, as demonstrated by both versions of the PIN7a reporter (P7A_1_G and P7A_2_G, respectively, Figure 3b), which show similar expression patterns in the root tip. This technique is, of course, limited in use only for monitoring protein-coding regions and practically not suitable, e.g., for assessing alternative polyadenylation or AS of non-coding RNAs.

In vivo sensors are commonly used in animal systems to demonstrate the developmental impact of AS. They have been used for the detection of tissue-specific expression of AS events e.g., in *C. elegans* [1,2,41], *D. melanogaster* [7], mouse [4,42] and human [3,5,6]. Moreover, these systems have also been instrumental in identifying the factors upstream of some of these AS events [2,3,5,6,7]. The usage of AS reporters in plant model organisms is scarce and has practically never been purposed for assessing cell-specific expression of splice isoforms [9,43,44]. To our knowledge, genetic screening with the marker, such as DAAO, has never been used in plants for such purpose at all. The fluorescent reporters can be used in the forward genetic screens as well. Exemplifying P7A_1_D, we even provide a more effective tool for the rapid and effective isolation of mutants with defects in this AS event.

An increasing number of pieces of evidence show that AS might be an important (and largely neglected) regulator of hormonal pathways, specifically in auxin-dependent processes [10,16,18,19,20]. The differential PIN7 AS reporter expression in columella following auxin treatment brings another interesting hint into these schemes. Altogether, our technological improvements can be therefore highly useful for plant biologists studying the post-transcriptional molecular mechanisms in hormonal and developmental pathways.

## 4. Materials and Methods

### 4.1. Plant Growth Conditions and Microscopy

All plant material was in *Arabidopsis thaliana* (L.) Heynh., Col-0 ecotype. Usually, the seeds were surface sterilized and, after 2 days of stratification in 4°C, cultivated under a 16 h:8 h photoperiod, 22:18°C, light:dark, on the 0.5 × MS medium [45] with 1% sucrose. For the reporter gene analyses, primary roots of 4–6 day-old vertically grown seedlings were used. Inducible transgene expression was controlled by the cultivation of the seedlings for six days (along with appropriate controls) on sterile media containing 5 µM 17-β-estradiol (est). The following chemicals were used for treatments: N^6^-benzyladenine (BA, diluted as 0.2 µM in DMSO), brassinolide (BL, 0.1 µM in DMSO), 1-naphthylacetic acid (NAA, 0.5 mM in DMSO), 17-β-estradiol (5 mM in DMSO), D-isoleucine (1 M in water), all from Sigma (Sigma-Aldrich, St. Louis, MO, USA). An equivalent amount of solvent was added to the control media.

The hormonal treatments were done under indicated conditions in the liquid 0.5 × MS medium supplemented with the appropriate compound and followed by the imaging. For examining the lines containing the *DAAO* transgenes, the seedlings were grown on the solid 0.5 × MS media supplemented with 30 mM D-isoleucine for 14–18 days and documented.

Confocal microscopy was conducted on inverted Zeiss Axio Observer.Z1 equipped with the standard confocal LSM880 and Airyscan modules with the 20×/0.8 DIC M27 air and 40×/1.2 W Kor FCS M27 air. For each experiment, 8 to 20 roots were analyzed in two independent biological replicates.

### 4.2. DNA Manipulations and Transgenic Work

The *AHP6:ahp6-2-mVenus* and *AHP6:AHP6-mCherry* constructs were made using the Multisite Gateway procedure (Invitrogen, Life Technologies, Carlsbad, CA, USA), inserted into the pHm43GW,0 vector as described [46,47], respecting the design of the original translational fusion [21]. The D4.1G.2R reporter was made using the Gibson Assembly method [48] (New England Biolabs, Ipswich, MA, USA) by recombination of the 2032-nucleotide sequence upstream of the start codon to the last coding codon of the isoform *DWF4.2* (1)*, RFP* with a stop codon (2) and the last intron plus last exon of the *DWF4.1* isoform (3), all inserted into the pMDC83 [33] backbone containing GFP. PIN7A_2_G was made by the analogous procedure, as described previously [10]. Cloning of the P4AG reporter was done as for *PIN7* [10], the entry pDONR202 vector containing the genomic DNA-based *PIN4-GFP* [49] construct was modified by inverse PCR and recombined in the LR Gateway reaction with the pMDC7 destination vector [33]. The P7A_1_D coding polynucleotides were custom synthesized (Gen9, Ginkgo Bioworks, Boston, MA, USA), cloned into the pDONR221 P5-P2 vector and together with the *PIN7* promoter [10] in the pDONR221 P1-P5r vector recombined with the pH7WG destination vector [47] in the Multisite Gateway LR reaction. The validated binary constructs were transformed into *Arabidopsis* by floral dipping and T2 or T3 generation of transformants showing the respective signal examined.
